# The burden of disease in metachromatic leukodystrophy: results of a caregiver survey in the UK and Republic of Ireland

**DOI:** 10.1186/s13023-023-03001-z

**Published:** 2024-02-25

**Authors:** Sophie Thomas, Alexandra Morrison, Georgina Morton, Pat Roberts, Vivienne Clark, Jackie Imrie

**Affiliations:** 1 MPS Society, MPS House, Amersham, HP7 9LP UK; 2 Rare Disease Research Partners, MPS House, Amersham, HP7 9LP UK; 3 ArchAngel MLD Trust, 59 Warwick Square, London, SW1V 2AL UK; 4 MLD Support Association UK, Floor 5, Amphenol Business Complex, Thanet Way, Whitstable, CT5 3SB UK

**Keywords:** Metachromatic leukodystrophy (MLD), Burden of illness, Inborn errors of metabolism, Caregiver burden, Natural history, Gene therapy

## Abstract

**Background:**

Metachromatic Leukodystrophy (MLD) is a rare, autosomal recessive lysosomal storage disease characterised by the progressive loss of motor function and severe decline in cognitive function. Limited information is available on the burden MLD places on patients and their families and the medical and social support these patients need. Three UK-based MLD patient organisations commissioned an online survey, and follow-up semi-structured interviews to describe and quantify these burdens across MLD subtypes, stage of disease (including end of life) and treatment status (untreated, gene therapy or hematopoietic stem cell transplant [HSCT]).

**Results:**

A total of 24 patients were included in the study: thirteen late infantile (LI), six early juvenile (EJ), two late juvenile (LJ) and three adult onset (AO). Six patients had received gene therapy and one had received an HSCT. MLD patients receiving no disease modifying treatment bore a high symptom burden: 94% were wheelchair dependent, 88% required tube feeding, 88% were incontinent, 82% had lost their speech and all the children were either unable to attend education or needed specialist provision. Patients were reliant on numerous medical interventions and assistive equipment. All early-onset patients (LI and EJ) were wheelchair dependent, and tube fed, with all EJ patients having lost all speech. The caregiving responsibilities of parents impacted their employment, finances, relationships and health. Patients treated with gene therapy or HSCT were more mobile and were able to eat normally and two thirds of the children were able to attend mainstream school.

**Conclusions:**

The impact of illness that patients and their caregivers faced was extensive, and the level of care, amount of medication, number of hospital visits and educational support required were substantial. Financial constraints often brought about by inability to work also placed considerable strain on families. The study increases understanding of the burden of MLD on patients and their families, and the level of unmet need in the treatment of the disease.

**Supplementary Information:**

The online version contains supplementary material available at 10.1186/s13023-023-03001-z.

## Background

Metachromatic Leukodystrophy (MLD) is a rare, autosomal recessive lysosomal storage disorder caused by a reduction in the enzyme arylsulfatase A (ARSA) and characterised by the accumulation of sulfatides in both the central nervous system and peripheral nervous system [1–3]. Although age of onset and initial symptoms vary, all patients eventually suffer a complete loss of motor, sensory and cognitive function, ultimately leading to premature death [2].

MLD is one of the most common leukodystrophies, and whilst the true worldwide prevalence rate is unknown, it is estimated to be between 1 in 40,000 and 1 in 160,000 [2, 4, 5]. In the UK, the incidence rate is estimated at 1 in 40,000 live births [5]. In general, MLD diseases occur in all ethnicities, although higher incidences are found in consanguineous populations such as the Habbanite Jews in Israel (1:75), Israeli Arabs (1:8000), Eskimos (1:2500) and Navajo Indians (1:6400) [2].

The clinical phenotype of MLD is heterogenous and patients may present with a broad range of neurological symptoms [3]. Eventually, the build-up of sulfatides causes a progressive loss of gross motor function, muscle spasms, seizures, rapid decline in cognitive function, loss of speech and incontinence [1].

The most common subtype, late infantile (LI) MLD presents in the first two years of life [2, 6, 7]. Children typically develop symptoms after an initial period of normal development, these include gait abnormalities, muscle weakness and developmental delay [2]. This form of MLD is considered the most severe, with rapid disease progression and death usually occurring between 5 and 8 years of age [4, 8]. Juvenile MLD is often divided into early juvenile (EJ) and late juvenile (LJ) forms. EJ MLD accounts for roughly a quarter of MLD cases and is characterised by the development of symptoms from the age of three years old [2, 6–8]. Disease progression is less rapid than in LI but nevertheless the same disease course follows, with death occurring within 10 to 20 years [2, 6–8]. LJ MLD presents at a later age, typically around the age of puberty, with behavioural issues ensuing first [2]. Adult onset (AO) is the rarest form of MLD, disease progression is far slower than early onset subtypes and the gradual decline in cognitive abilities may be difficult to identify [7].

Until recently, palliative care was the only treatment option in MLD for most patients, including drugs to provide pain relief, control seizures and treat infections [9]. Haematopoietic stem cell transplantation (HSCT) has appeared beneficial if administered early in late-onset patients, but clinical data revealed significant limitations [10]. Recently, a gene therapy (atidarsagene autotemcel) for the treatment of LI or EJ forms, without clinical manifestations of MLD, was approved by the European Medicines Agency in 2020 [11]. In 2022, atidarsagene autotemcel was recommended as a treatment option by the National Institute for Health and Care Excellence for eligible children in England/Wales and granted Scottish Medicines Consortium (SMC) approval for eligible children in Scotland under the ultra-orphan pathway [12].

Although some data has been published on MLD caregiver quality of life and the impact on families caring for a loved one with MLD [13, 14], limited data on the disease burden experienced by patients and their quality of life are available [19]. These data are needed as potential new treatments are developed and assessed in Health Technology Appraisals (HTAs). For this reason, a study was commissioned by three patient organisations, the MPS Society, the MLD Support Association UK and the ArchAngel MLD Trust, all of which support patients and their families with MLD in the UK. The study had two aims. One was to gather information relevant to the consideration of MLD as a candidate for newborn screening. The results from this part of the study have been published [15]. The other aim of the study was to describe and quantify the burden MLD places on patients and their families and some wider impacts on medical, educational and social services. The results of this part of the study are presented here and include the burden on patients across key domains including symptoms and treatment burden. In addition, the caregiving burden on families is presented in terms of the time spent providing care and the financial, social, physical and emotional impacts. The support patients and families require in education, care provision, medical equipment and home adaptations are also presented.

## Methods

### Study design

The study was cross-sectional and employed a mixed methods approach with an online survey and semi-structured interviews. This design was chosen to allow for the patient and caregiver burden to be quantified (via the survey) and its impacts to be described in more depth than a survey methodology allows, via the interviews. The patient organisations who commissioned the study wanted to give the participants a strong voice to share their experience and mixed methods can facilitate this and also enable the research questions to be answered in more depth [16].

The online survey used a specifically designed questionnaire with input from patient experts from the three organisations covering demographics, overall symptom burden, hospital visits, medication and surgical interventions, impact on parent’s ability to work, and home adaptations. Questions were presented as multiple choice where possible, with free text to include additional information not covered by the answer options. The online surveys were completed between 28 August and 18 October 2020. In the online survey, respondents were asked to indicate the presence or absence of symptoms at various time points to gain an understanding of the progression of MLD and for deceased patients, respondents were asked about the patient’s symptoms at the final stage of disease. Respondents who had completed the online survey were eligible to take part in the in-depth semi-structured interviews. A semi-structured interview guide was developed, designed to explore further the items raised in the online survey. Interviews were all conducted over the telephone with the same member of the MPS Society’s patient services team and took place between 29 September and 21 October 2020. Calls were audio recorded and transcribed for thematic analysis. Analysis of the online survey results and interview transcripts was undertaken by Rare Disease Research Partners. This research was conducted in accordance with the British Healthcare Business Intelligence Association’s Legal & Ethical Guidelines for Market Research [17].

### Patient selection

Members of three patient organisations, the MPS Society, the MLD Support Association UK and the ArchAngel MLD Trust were invited by email and telephone to participate in this study. The results reported here are part of a larger study, that included an examination of disease progression and caregiver views on newborn screening which have been reported elsewhere.

### Inclusion criteria

To be eligible, participants had to be a patient, the parent or main caregiver of a live or deceased person with a confirmed diagnosis of MLD, aged ≥ 18 years and a resident of the UK or Republic of Ireland. Only participants able to provide informed consent to participate were included. Parents or a caregiver with more than one child with MLD, were asked to complete a separate questionnaire (and interview, if applicable) for each child. The definitions used for the MLD clinical subtypes were:

- Late infantile (symptom onset ≤ 2.5 years of age).
- Early juvenile (symptom onset > 2.5 to < 7 years of age).
- Late juvenile (symptom onset 7 to < 17 years of age).
- Adult onset (symptom onset ≥ 17 years of age).

## Results

### Demographics

Twenty-four responses to the survey were received and most respondents were parents of patients that were alive at the time of the survey (n = 21), with the remaining consisting of bereaved parents (n = 2) and one bereaved caregiver). Patients were from 20 families (two families had two children with MLD, one family had three children with MLD). After completing the survey, six parents took part in the interviews, including the three families with more than one child with MLD, giving a total of ten individuals with MLD. In total, 58% (n = 14) were female and 88% (n = 21) were from England, with the remaining patients from the Republic of Ireland (8%, n = 2) and Northern Ireland (4%, n = 1). Thirteen patients had LI MLD, six had EJ MLD, two had LJ MLD and three had AO MLD (Table 1). Three of the LI patients and three of the EJ patients were treated with gene therapy and of the three AO patients, one patient had received an HSCT 25 years ago (Table 1). The mean age of patients (n = 21) at the time of the survey was 12.3 years, range 2—48 years, and the median age of patients was 7 years. Three patients were deceased, the age at death ranged from 5 to 39 years. These patients were born between the years 1962—2012.

**Table 1:** Patient characteristics

Patient characteristics	Late infantile (N = 13)	Early juvenile (N = 6)	Late juvenile (N = 2)	Adult onset (N = 3)
Patients receiving no disease modifying treatment	10	3	2	2
Alive, n	8	3	2	1
Deceased, n	2	0	0	1
Age now	5.6 (2–7)	13.3 (11–16)	24.0 (15–33)	30
Age at death	6.3 (5–7.5)	-	-	39
Patients receiving gene therapy or HSCT^a^	3	3	0	1
Alive, n	3	3	0	1
Deceased, n	0	0	0	0
Age now	4.7 (4–5)	11.3 (9–13)	-	48
Age at death	-	-	-	-

Data are mean (range), years unless specified

*HSCT = haemopoietic stem cell transplant*

^a^ Only one patient in the adult onset subgroup received HSCT, all other patients received gene therapy

### Burden of Illness in patients receiving no disease modifying treatment

#### Overall symptom burden in LI patients

The mean age of symptom onset in LI patients was 1.5 years and initial symptoms included trouble walking, musculoskeletal problems, and difficulty swallowing. In the interviews, all three parents described how issues with walking had been one of the first signs that something was wrong. None of these patients had progressed to walking unaided and all had become wheelchair dependent during the course of their disease. 80% of patients had lost the ability to speak and 60% were no longer able to communicate pain or discomfort (Supplementary Table 1).

While most patients met their early learning developmental milestones, they required specialist education and support in learning from an early age. Often, they were only able to attend a specialist school for a short period of time before the need to move to home schooling or they became too ill to continue their education (Supplementary Table 1).

Musculoskeletal issues were common in LI patients, with dystonia and hypertonia reported in 80% of patients (Supplementary Table 1). A parent of a child with LI MLD described the physical transformation of their child over time:

His body has changed the most. So he’s got two dislocated hips. He’s got curvature of his spine. He obviously has spasms and jerks and dystonia. His weight has never really gained, so he’s very, very tiny. He’s very long, so growth has still continued. There’s kind of no resemblance, really, to what was there before. And also he can’t use, he doesn’t enjoy using, his hands are closed a lot of the time. He can’t even use his hands to press a switch toy or anything like that.

Eyesight and hearing deteriorated as the disease progressed. In the final stages of disease, patients may be blind and deaf. Earlier on it may be difficult to determine how much sight and hearing is retained. Neurological symptoms such as seizures, anxiety and panic were prevalent (Supplementary Table 1).

All LI patients had progressed to tube feeding, with 90% of patients fitted with a gastronomy tube and 10% fitted with a nasogastric tube (Supplementary Table 1).

Some parents mentioned the occurrence of vomiting. In the online survey, one patient was described as experiencing vomiting and diarrhoea 10—15 times a day in the final stages of MLD. In the interviews, one parent talked about vomiting including episodes that required hospital treatment. Constipation, incontinence, and urinary retention were common, with half of LI patients being doubly incontinent (Supplementary Table 1).

Many LI patients suffered with chest and respiratory problems. One parent described their child’s difficulty breathing:

It feels really awful, because when he’s really struggling [to breathe] your heart bleeds, because you’re thinking, I know he’s finding it really hard. And we do everything. We’ve got special neck rolls, cushions that we put under his neck to make sure that his neck and his jaw are supported as much as possible. We pinch his jaw when he’s struggling.

The effects of MLD on the muscles and joints caused a great deal of pain, and it was often difficult to identify the source. Patients may also experience neuropathic pain and 80% percent were taking pain relieving medication:

Like he has overall systemic sort of nerve pain and discomfort, but he doesn’t have any specific pains in his hands and feet. So I suppose he had his first dislocated hip, and that was a long period and very painful for him. And then the second one dislocated, and that was, again, another long two or three months of pain before that one came out.

Parents also talked about sleep disturbance associated with pain:

She would take a long time to fall asleep and she would cry a lot as well. She was in pain, but it wasn’t obvious where she might be in pain.

#### Overall symptom burden in EJ and LJ patients

The mean age of symptom onset in EJ patients and LJ patients was 5.3 years and 10.5 years, respectively. The most common presenting symptoms were issues with walking, learning and behaviour. All EJ patients attended specialist school, one parent reported a rapid decline in the child’s learning during the six-month period from first symptoms to diagnosis:

And she’d always loved writing as well. She was forever making lists. And she was quite a bright academic little girl. She just wasn’t interested anymore. She wasn’t interested in her reading anymore and she absolutely devoured books. She loved them.

Another child was wheelchair bound, tube fed, unable to communicate verbally or non-verbally and blind. The child’s parent described the type of activities that the school offered in the one-to-one support provided:

Lots of sensory stuff, really. Yesterday, they had all the lights on in the sensory room. They do something they call Tacpac, which I don’t… I’m sure there’s a really good reason and understanding behind it, but they put sponges up their legs and just tactile type stuff, I suppose. Very sensory based. Lots of fun games. Lots of dancing. She does wheelchair dancing.

Both LJ patients reported learning issues as initial symptoms and experienced confusion, disorientation and problems concentrating (Supplementary Table 2).

Some EJ patients lost the ability to speak but were able to communicate non-verbally for a short time, One child had mastered an eye communication device really quickly, but after 6 months was no longer able to use it. One LJ patient had deteriorating speech and the second had lost the ability to speak and communicate non-verbally (Supplementary Table 2). Most EJ and LJ patients were blind, one parent described the rapid decline in their child’s eyesight:

I feel it’s really quite obvious that there’s been a massive decline in her eyesight. She doesn’t look at you anymore when you’re talking. She used to follow you around the room. Or if you were stood in a corner of the room and you talk, she would look that way. Her eyes would move that way. But her eyes just didn’t seem to focus on anything anymore.

During the course of their disease, all EJ and LJ patients became wheelchair dependent (Supplementary Tables 1 and Supplementary Table 2). All EJ patients were fed by gastronomy tube and one LJ patient was fed by nasogastric tube. One parent of an EJ patient described how their child suffered with severe vomiting:

She was sick all the time, constantly morning, noon, night, through the night. She was just constantly sick. We’d get up in the morning and there will be a pile of washing from the night before. And it could have been just been a couple of towels [?] and we’d been lucky to catch it or it could have been the whole bedding and pajamas and duvets and everything.

EJ patients were doubly incontinent, all having suffered with constipation and one with urinary retention, whereas one LJ patient was doubly incontinent, had urinary retention and constipation (Supplementary Tables 1 and Supplementary Table 2). Aspiration, excess secretions, and frequent chest infections were also experienced (Supplementary Tables 1 and Supplementary Table 2). One parent of an EJ patient explained the consequences of excess secretions:

The biggest thing that she has issues with now is secretions. She failed the oxygen levels. We’re just in the process this week of getting some oxygen for her to help overnight. And I think the biggest cause of that is secretions. And you can be up in the middle of the night doing that. All these things just try to clear it so she’s getting… She’s able to breathe more easily. That is certainly over the last six months, I would say, has been the biggest issue that’s got worse. The secretions and the oxygen levels.

The turmoil of living with MLD was expressed by parents of a child suffering from the EJ form:

In a nutshell, it’s destroyed our lives really. It has destroyed our lives completely and utterly. Not just [Name’s], but ours as well because we’ve had to watch it and there’s really not much we can do really. And probably extended family as well, grandparents. It’s not just us, not just me and [Name]. I think grandparents as well. It’s destroyed their lives as well, really….She’s been tortured, basically. She is. That’s what the disease is doing to her. It’s torturing her little body. And we had to sit and watch that. We have to sit and watch it. And other than cuddling her and giving her meds, there’s just nothing we can… And I’d swap places with her.

#### Overall symptom burden in AO patients

The mean age of symptom onset in AO patients was 25 years, initial symptoms presented as a change in behaviour and cognitive deterioration. Both adult patients had changes in behaviour, confusion/disorientation, learning, memory, sleep disturbance and concentration issues as early signs of disease (Supplementary Table 2). One patient had difficulty with co-ordination, whilst the other was wheelchair dependent. Both patients had lost the ability to speak and communicate verbally. One had lost speech by the time of diagnosis and the other was repeating certain phrases at that time (Supplementary Table 2). Disease progression was obvious in one adult patient in the lead up to diagnosis and described by the parent:

If we’d got the diagnosis a year earlier, he would probably have been living independent life still, albeit supported. Because it was that last year, was really when the symptoms started to manifest. And it was obvious we couldn’t leave him alone for any length of time. We had to monitor what was happening. He’d put a meal in the oven to cook and then go out.

### Medical needs

The mean number of hospital outpatient visits and length of hospital stays in the last 12 months was far greater in LI and EJ patients than in LJ and AO patients (Supplementary Table 3). Most LI patients had between 2 and 10 hospital outpatient visits, although for one patient there were more than 100 visits.

All patients required a variety of medications including anti-secretion medication, anti-seizure medication, muscle relaxants and pain medication (Supplementary Table 4). All patients needed a range of interventions including oxygen, suctioning and catheters. One LJ patient needed both suctioning and a urinary catheter (Supplementary Table 4). The fitting of gastrostomy tubes was more common in LI and EJ patients than in LJ and AO patients. One LI patient had the tendons in the ankles cut to relieve pain and one LJ patient had undergone surgery for scoliosis (Supplementary Table 4).

### Caregiver burden

For LI patients, two mothers spent around 100 h per week caring for their child and the remaining six mothers described the care as 24 h per day, 7 days a week. This was also the case for half of the fathers. Two families had support from another family member for a few hours per week. Eighty percent of LI patients received additional care support from professional carers, hospices and respite care (Supplementary Table 5). MLD also greatly impacted both the mother’s and father’s ability to work from when the children were around two years old. Some had to reduce hours or find more accommodating jobs before finally having to leave work.

The care burden was also significant in juvenile patients, with mothers of EJ spending a mean of 56 h a week caring for their child and fathers spending a mean of 46 h. All EJ patients required professional care and one patient needed 45 h of support from a night carer per week (Supplementary Table 5). Both LJ patients needed full-time care involving the parents and professional carers and one patient needed two carers for moving. MLD had a substantial impact on both parents’ ability to work with at least one parent having to give up work to provide care for their child. The mother of an LJ patient had to change her job which resulted in a loss of income and her husband was made redundant and was left unable to find suitable employment. The parents of all MLD patients needed to rely heavily on benefits to cope financially (Supplementary Table 5).

Relationships with the wider family and friends often suffered from break down and siblings had to take second place to the child with MLD and may have to become caregivers themselves. Siblings experience grief and loss as their brother or sister loses their abilities and they see them suffer:

*[Brother] has grieved for the loss of [name’s] skills. He openly talks about how he wishes [name] could play with him again and how he is sad that he can’t walk or talk. [Brother] has been witness to [name] being in a lot of pain with dystonia and with violent sickness.*

Siblings may have to take second place: *Worry, guilt, not always able to go places that they would like, separation from parents when siblings are ill or need care, have to grow up quickly. Majority of time have to take 2nd place.*

Their lives are affected to some extent as hospital appointments, medication and the comfort of their sibling has to take priority.

Parents also described a range of impacts on their health and wellbeing. Most had back pain due to moving their child and mental health issues such as stress, anxiety, grief, depression, and isolation were common.

### Home adaptations and equipment

Home adaptations were required for 80% of LI patients and for 100% of EJ and LJ patients. The most common alteration needed for LI patients was bathroom adaptation, whereas access adaptations and hoists were additional requirements for juvenile patients (Supplementary Table 6). One family of a child with LJ MLD had to move to more suitable accommodation to provide adequate care for their child. For one of the AO patients, no home adaptations or equipment were necessary as they deteriorated quite quickly after diagnosis and went into a nursing home and the other family moved to more suitable accommodation (Supplementary Table 6).

### Overall symptom burden, medical needs, caregiver burden and home adaptations in patients receiving gene therapy or HSCT

Overall, there were three LI patients and three EJ patients that underwent gene therapy. Of the LI patients, two children (twins) received gene therapy aged between 8 and 10 months, the third child was treated at 1 year of age. These children were all asymptomatic and received their diagnosis due to diagnosis of MLD in older siblings and are now all aged between 4 and 5 years (Table 1). The three EJ patients received gene therapy aged between 4 and 7 years and are now aged between 9 and 13 years (Table 1). One AO patient received an HSCT at 23 years of age, three years after symptom onset and is now 48 years old.

LI children treated with gene therapy had very few symptoms and were all able to attend mainstream school (Supplementary Table 6), one parent described how well their child was doing:

He’s doing well. He recently had a kind of a cognitive assessment and we were really delighted to see he’s within normal sort of ranges for everything.

Two of the EJ patients that had issues with walking prior to treatment became wheelchair dependent over time and the remaining patient had no mobility issues and leads an active life:

He was going to a football club, but he didn’t really like it. He loves swimming. He’s done the 21-mile, walked 21 miles and made some money for charity.

Some musculoskeletal and neurological symptoms were present in EJ patients, with all three patients suffering from hypotonia and two patients experiencing temperature dysregulation, sensory processing issues and uncontrollable crying (Supplementary Table 6). The AO patient treated with an HSCT had no mobility, speech, or musculoskeletal issues and seizures were the only neurological symptom reported. The patient suffered with cognitive symptoms, which were present prior to HSCT.

Of the three LI patients that underwent gene therapy, only one patient recorded hospital outpatient visits. The number of outpatient visits were also low in EJ patients, with a mean of three outpatient visits per patient in the last 12 months. Gene therapy patients required minimal medication, interventions, or surgery. One EJ patient required anti-secretion medication and laxatives, one EJ patient needed a physiotherapy vibration vest to loosen secretions and one patient required surgery. The AO patient that underwent HSCT only required anti-seizure medication.

LI patients treated with gene therapy only required the “typical” amount of care to raise a family, whereas all EJ patients needed extra care by mothers. In one of the gene therapy families both parents had given up work when both of their children had been diagnosed with MLD. One parent of an EJ patient that underwent gene therapy described her change of career and how she was able to work again following improvements in her child’s condition after treatment. The AO patient that underwent an HSCT returns home most weekends with both parents spending an average of 24 h per week caring for their child. One EJ patient treated with gene therapy required home adaptations such as a hoist and bathroom adaptation and one LI patient moved to more suitable accommodation. A range of equipment was required for several of the LI and EJ patients treated with gene therapy including a wheelchair, toileting chair, sleep system and sensory toys. No home adaptation or equipment was needed for the HSCT patient.

## Discussion

Owing to the rare nature and severity of MLD, limited data on the impact of the disease are available. In this study, qualitative analysis of parental and caregiver accounts provided novel insights into the burden of illness faced by patients with MLD and their families. Our study collected information including overall symptom burden, medical needs, caregiver burden and home adaptations required. Due to the variability of symptoms in MLD and their crossover with other conditions, MLD is often misdiagnosed or diagnosed late, after significant tissue damage has occurred [7]. In early-onset forms (LI and EJ), rapid disease progression is observed and without therapy death occurs within a few years of disease onset [18]. Our study revealed that patients receiving no disease modifying treatment suffered from a great range and severity of symptoms. MLD patients became wheelchair dependent and lost the ability to speak and communicate non-verbally during the course of disease. These patients required numerous medications, surgical interventions, and home adaptations. Our study showed that early-onset patients that underwent gene therapy were able to enjoy a more normal life, including some being able to attend mainstream school.

Our data clearly demonstrate the severe impact of disease burden faced by the patient and families. The variety and complexity of symptoms have a resounding effect on the family in terms of their financial, emotional, and social status. Mobility was lost early in the disease course, which changed the daily life of the parent or caregiver profoundly. This often required significant adaptations to the home, with all three EJ patients receiving no disease modifying treatment requiring a hoist to be installed. A similar study conducted in 2016, interviewed 30 caregivers of 23 MLD patients and reported the most frequent symptoms [14]. This study included patients across a range of subtypes (14 Late infantile, 6 juvenile and 3 adult onset) from the United States (US), Columbia, France, and Germany. The most common cognitive symptoms described were difficulty understanding/ processing information (33%), general cognitive problems (27%), and lack of awareness (20%). Caregivers also depicted behavioural changes in patients such as aggressiveness, loss of inhibition, lack of judgment/responsibility, withdrawal/disorientation, and abnormal behaviour. Physical symptoms frequently reported by caregivers across all subtypes were: difficulty in walking or stopped walking/crawling (77%), difficulty swallowing (50%), seizures (50%), poor vision (47%), loss of motor skills (43%), breathing/respiratory problems (40%), muscle tension/stiffness (40%), pain (37%) and balance (33%) [14]. These symptom data incorporate all MLD subtypes, and although most symptoms described support the findings of our study, we found a higher percentage of patients were wheelchair dependent or immobile at the time of the survey (100% of LI, EJ, and LJ patients receiving no disease modifying treatment). A US study of 32 caregivers representing 16 LI patients and 16 juvenile patients found that 87.5% of LI patients had little or no functional movement or had died having lost all gross motor function [1]. In our study, we reported numerous hospital visits in patients receiving no disease modifying treatment, the mean number of hospital outpatient visits in a 12-month period for LI patients and EJ patients was 18 and 14, respectively. In the 2016 study, caregivers reported frequent hospitalizations, with 27% patients having experienced ≥ 11 hospitalizations since diagnosis [14].

As the disease progressed, more financial strain was placed on the family and the number of hours spent caring for children also affected the ability of parents to work. The progression of MLD draws parallels with the disease course of other lysosomal storage disorders (LSDs) such as MPS and Battens disease [19–23]. The associated loss of motor function leads to a high dependence on physical care, with needs changing as symptoms develop [24]. Here, we found that almost all mothers of LI patients receiving no disease modifying treatment spent more than 100 h caring for their child per week. A recent study reported the time spent caring for a child with an LSD ranged from 8.6 to 16.6 h a day on average [25–27]. The same study also reported a strong correlation between the severity of the condition, the impact on family and quality of life [24]. Our data showed a clear impact on parents’ working lives, with many forced to reduce their working hours, change job, or leave their job entirely in order to provide care for their child.

Although our study sample was small, the number of patients included represented a large proportion of the total number of MLD patients known to patient groups in the UK and Ireland (23 paediatric, 6 adult and 19 deceased). Due to the study design, comparisons between the patients receiving no disease modifying treatment and those receiving gene therapy could not be made. Most patients presented with early-onset forms of MLD, which are the most common. Important insights on overall symptom burden, medical needs, caregiver burden and home adaptations needed were ascertained through the use of a mixed methods approach, which allowed issues to be explored in detail and new ones identified. As the study relied on caregiver reports it had potential limitations, such as the requirement for parents and caregivers to remember symptoms, medications, and other aspects of care retrospectively. There was also some variability in the data available for each patient as not all respondents answered all questions in the survey. Regardless of these limitations, our study captures a broad range of data and reinforces the multitude of symptoms that patients with MLD encounter, and the significant burden placed on the family.

## Conclusions

The parental and caregiver descriptions of the impacts of MLD indicate a severe burden of disease. The level of care, amount of medication, number of hospital visits and educational support required were extensive. The LI and EJ forms progress rapidly and lead to a total loss of function including immobility, loss of all communication, dementia, painful issues involving the skeleton, muscles and joints including dystonia and hip dislocations, neurological problems, blindness, deafness, incontinence and the need for tube feeding. The LJ and AO forms of MLD may progress at a slower pace but ultimately follow the same path. Parents must not only deal with the deteriorating health of their child, but often become full-time carers and carry the burden of physical, mental, financial and social problems that this brings. Relationships with the wider family and friends often break down. Siblings have to take second place to the child with MLD and may become caregivers themselves. Families often have to rely on a range of benefits and need to adapt their homes and install specialist equipment to care for their child. Patients with AO MLD are unable to continue in employment or further education as their behaviour and cognitive decline makes it impossible. They may need residential care, particularly as their parents age.

The study increases understanding of the burden of MLD on patients and their families, and the level of unmet need in the treatment of the disease.

### Electronic supplementary material

Below is the link to the electronic supplementary material.

Supplementary Material 1

## Data Availability

In order to maintain patient confidentiality, raw data from this study is not available.

## Abbreviations

AO: Adult onset
ARSA: Arylsulfatase A
EJ: Early juvenile
HSCT: Hematopoietic stem cell transplant
LI: Late infantile
LJ: Late juvenile
LSDs: Lysosomal storage disorders
MLD: Metachromatic Leukodystrophy
SMC: Scottish Medicines Consortium
US: United States
