# Supplementary material for: The burden of disease in metachromatic leukodystrophy: results of a caregiver survey in the UK and Republic of Ireland
Source: Orphanet J Rare Dis. 2024 Feb 25;19:87. doi: 10.1186/s13023-023-03001-z (PMC10895743; doi:10.1186/s13023-023-03001-z)
Supplement: Supplementary file 1 — Supplementary Material 1 [file 13023_2023_3001_MOESM1_ESM.docx]

**Supplementary Tables**

**Supplementary Table 1. Overall symptom burden in untreated patients: Late infantile and early juvenile.**

| **Overall symptom burden**  **Frequency, n (%)** | **Late infantile**  **(N=10)** | **Early juvenile**  **(N=3)** |
| --- | --- | --- |
| Mobility  Wheelchair dependent or immobile  Walking with aids  Some issues with walking  No mobility issues | 10 (100)^a^  0  0  0 | 3 (100)  0  0  0 |
| Learning  Unable to attend school  Attend specialist school  Attend specialist school outreach  Homeschooled  Mainstream school | 2 (29)^b^  2 (29)^b^  1 (14)^b^  2 (29)^b^  0 | 0  3 (100)  0  0  0 |
| Speech and communication  Lost all communication^c^  Lost ability to speak  Speech deterioration  No speech issues reported | 0  8 (80)  0  2 (20) | 3 (100)  0  0  0 |
| Musculoskeletal  Dystonia  Hip subluxation or dislocation  Hypotonia  Scoliosis  Spasticity or hypertonia  No musculoskeletal issues | 8 (80)  4 (40)  6 (60)  4 (40)  8 (80)  2 (20) | 3 (100)  2 (67)  3 (100)  3 (100)  3 (100)  0 |
| Eyesight and hearing  Blindness  Vision difficulties  Deafness  No issues with eyesight or hearing | 3 (30)  3 (30)  2 (20)  3 (30) | 3 (100)  0  0  0 |
| Neurological symptoms  Anxiety or panic  Temperature dysregulation  Peripheral neuropathy  Seizures^d^  Sensory processing issues  Sleep disturbance  Uncontrollable crying  No neurological symptoms | 6 (60)  5 (50)  3 (30)  8 (80)  4 (40)  5 (50)  5 (50)  2 (20) | 2 (67)  3 (100)  2 (67)  2 (67)  2 (67)  2 (67)  1 (33)  0 |
| Nutrition and eating  Fed by nasogastric tube  Fed by gastronomy tube  Gall bladder issues  No feeding or gallbladder issues | 1 (10)  9 (90)  5 (50)  0 | 0  3 (100)  2 (67)  0 |
| Chest and respiratory symptoms  Aspiration  Excess secretions^e^  Frequent chest infections  Frequent colds or runny nose  No chest or respiratory symptoms | 5 (50)  8 (80)  3 (30)  3 (30)  1(10) | 3 (100)  3 (100)  3 (100)  2 (67)  0 |
| Bowels and bladder  Constipation  Urinary retention  Urinary and bowel incontinence  Urinary incontinence only  No bowel or bladder issues | 7 (70)  6 (60)  5 (50)  1 (10)  3 (30) | 3 (100)  1 (33)  3 (100)  0  0 |

^a^ Includes one patient where respondent answered ‘no mobility issues’ but later in the questionnaire referred to parents having bad backs due to lifting and moving child, having a bathroom adapted and using a wheelchair or specialist buggy.

^b^ Total number of 7 live school-aged children.

^c^ 60% had lost ability to articulate pain or discomfort, 50% had lost ability for non-verbal communication.

^d^ Suffering from seizures or are on anti-seizure medication.

^e^ Suffering from excess secretions or are on anti-secretion medication.

**Supplementary Table 2. Overall symptom burden in untreated patients: Late juvenile and adult onset.**

| **Overall symptom burden**  **Frequency, n (%)** | **Late juvenile**  **(N=2)** | **Adult onset**  **(N=2)** |
| --- | --- | --- |
| Mobility  Wheelchair dependent or immobile  Some issues with walking  No mobility issues | 2 (100)  0  0 | 1 (50)  1 (50)  0 |
| Learning (cognitive symptoms)  Learning issues  Memory and concentration issues  Confusion or disorientation  Dementia  No cognitive symptoms | 2 (100)  2 (100)  2 (100)  1 (50)  0 | 2 (100)  2 (100)  2 (100)  1 (50)  0 |
| Speech and communication  Dysarthria  Dysphasia  Lost all communication  Lost ability to speak  Speech deterioration  No issues with speech | 1 (50)  1 (50)  1 (50)  1 (50)  1 (50)  0 | 0  2 (100)  2 (100)  2 (100)  0  0 |
| Musculoskeletal  Dystonia  Hip subluxation or dislocation  Hypotonia  Scoliosis  Spasticity or hypertonia  No musculoskeletal issues | 2 (100)  1 (50)  1 (50)  1 (50)  2 (100)  0 | 1 (50)  1 (50)  1 (50)  1 (50)  1 (50)  1 (50) |
| Eyesight and hearing  Blindness  Deafness  No issues with eyesight or hearing | 1 (50)  0  1 (50) | 1 (50)  1 (50)  1 (50) |
| Neurological symptoms  Anxiety or panic  Temperature dysregulation  Peripheral neuropathy  Seizures or epilepsy  Sensory processing issues^a^  Sleep disturbance  Uncontrollable crying  No neurological symptoms | 1 (50)  1 (50)  2 (100)  1 (50)  2 (100)  2 (100)  1 (50)  0 | 1 (50)  1 (50)  2 (100)  2 (100)  1 (50)  1 (50)  2 (100)  0 |
| Nutrition and eating  Difficulty swallowing/risk of choking  Fed by nasogastric tube  Gall bladder issues  No feeding or gallbladder issues | 0  1 (50)  0  1 (50) | 1 (50)  1 (50)  1 (50)  0 |
| Chest and respiratory symptoms  Aspiration  Excess secretions  Frequent chest infections  Frequent colds or runny nose  No chest or respiratory symptoms | 1 (50)  1 (50)  1 (50)  1 (50)  1 (50) | 0  0  0  0  0 |
| Bowels and bladder  Urgency/frequent accidents  Constipation  Urinary retention  Urinary incontinence  Urinary and bowel incontinence  No issues with bowels and bladder | 0  1 (50)  1 (50)  0  1 (50)  1 (50) | 1 (50)  0  1 (50)  1 (50)  0  1 (50) |

^a^ Including light sensitivity.

**Supplementary Table 3. Mean number of hospital visits or stays connected to MLD in the last 12 months in untreated patients.**

| **Mean number of hospital visits or stays** | **Late  infantile (N=8)^a^** | **Early juvenile**  **(N=3)^a^** | **Late  juvenile (N=2) ^a^** | **Adult  onset**  **(N=1) ^a^** |
| --- | --- | --- | --- | --- |
| Number of hospital outpatient visits | 18 | 14 | 3 | 2 |
| Number of hospitalisations | 3 | 1 | 0.5^b^ | 0 |
| Total length of stay in hospital (days) | 15 | 11 | 0.5^b^ | 0 |

^a^ Number of live patients.

^b^ One patient was hospitalised for one day.

**Supplementary Table 4. Medications, interventions, and surgery required for MLD management in untreated patients.**

| **Medicines, interventions, and surgeries**  **Frequency, n (%)** | **Late  infantile (N=10)** | **Early juvenile**  **(N=3)** | **Late  juvenile (N=2)** | **Adult  onset**  **(N=2)^a^** |
| --- | --- | --- | --- | --- |
| Medicines^a^  Patients requiring medicines  Anti-secretion medication  Anti-seizure medication  Medicine to manage digestive issues  Muscle relaxants for spasms  Pain medication  Other | 8 (100)  7 (88)  6 (75)  5 (63)  7 (88)  7 (88)  1 (13)^b^ | 3 (100)  3 (100)  2 (67)  3 (100)  3 (100)  3 (100)  0 | 2 (100)  1 (50)  1 (50)  2 (100)  2 (100)  1 (50)  0 | 1 (100)  0  1 (100)  0  0  0  1 (100)^c^ |
| Medical interventions^a^  Patients requiring interventions  CPAP or BI PAP  Brace for scoliosis  Enemas for constipation  Suctioning  Urinary catheter  Other | 7 (88)  1 (13)  0  2 (25)  6 (75)  3 (38)  1 (13)^d^ | 2 (67)  0  1 (33)  1 (33)  2 (67)  1 (33)  1 (33)^e^ | 1(50)  0  0  0  1 (50)  1 (50)  0 | 0  0  0  0  0  0  0 |
| Surgeries  Patients requiring surgeries  Gall bladder removal  Gastronomy tube  Surgery for hip dislocation  Other | 9 (90)  2 (20)  9 (90)  1 (10)  1 (10)^f^ | 3 (100)  0  3 (100)  0  1 (33)^g^ | 1 (50)  0  0  0  1 (50)^h^ | 0  0  1 (100)^i^  0  0 |

^a^ Questions asked for live patients only.

^b^ Clonidine and clonazepam.

^c^ Patient receives desmopressin (vasopressin analogue) and mirtazapine (anti-depressant).

^d^ Nebuliser, splints, and oxygen.

^e^ Ankle/foot orthoses.

^f^ Tendons in ankles cut.

^g^ Tendon repair to facilitate weight bearing.

^h^ One patient had surgery for scoliosis.

^i^ One respondent did not answer this question.

**Supplementary Table 5. Care burden in untreated patients.**

| **Care burden**  **Frequency, n (%)** | **Late  infantile (N=10)** | **Early juvenile**  **(N=3)** | **Late  juvenile (N=2)** | **Adult  onset**  **(N=2)** |
| --- | --- | --- | --- | --- |
| Family care  Care by mothers  Care by fathers  Care by other family member | 10 (100)^a^  5 (50)^a^  2 (20)^a^ | 3 (100)^b^  3 (100)^c^  0 | 2 (100)  1 (50)  0 | 0  0  0 |
| Additional care  Professional carer  Social worker  Residential care  Weekend carer  Night-time support  Hospice  Respite care  No support needed  Refused support  Other | 8 (80)  4 (40)  0  0  0  0^f^  7 (70)  3 (30)  1 (10)  1 (10)  0 | 3 (100)  3 (100)  2 (67)  0  0  0  2 (67)  2 (67)  0  0  0 | 2 (100)  2 (100)  2 (100)  0  0  0  0  0  0  0  1 (50)^d^ | 2 (100)  1 (50)  0  2 (100)^e^  0  0  0  1 (50)  0  0  0 |
| Benefits claimed  Carers allowance  Child tax credit  Disability living allowance  Employment support  Housing benefit  Universal credit  Working tax credit  Council tax benefit  Residential care package  Personal independence payment | 9 (90)  8 (80)  5 (50)  9 (90)  1 (10)  2 (20)  2 (20)  2 (20)  0  0  0 | 3 (100)  2 (67)  1 (33)  3 (100)  0  1 (33)  0  0  0  0  0 | 2 (100)  1 (50)  0  1 (50)  0  0  0  0  1 (50)  1 (50)  1 (50) | 2 (100)  2 (100)  0  1 (50)  1 (50)  0  0  0  0  1 (50)  1 (50) |

^a^ Over 100 hours of childcare per week.

^b^ Mothers spent between 40-88 hours per week of childcare per week.

^c^ Fathers spent between 20-88 hours per week of childcare per week.

^d^ Play worker, occupational therapist, physiotherapist and NHS Continuing Health Team.

^e^ The live patient was cared for at home for two years before moving to residential care and the deceased patient was in residential care.

**Supplementary Table 6. Home adaptations and equipment in untreated patients.**

| **Home adaptations and equipment required**  **Frequency, n (%)** | **Late  infantile (N=10)** | **Early juvenile**  **(N=3)** | **Late  juvenile (N=2)** | **Adult  onset**  **(N=2)** |
| --- | --- | --- | --- | --- |
| Home adaptations needed | 8 (80) | 3 (100) | 2 (100) | 0 |
| Equipment needed | 10 (100) | 3 (100) | 0 | 0 |
| Bathroom  Bathroom adaptation  Bath chair  Toileting chair | 4 (40)  9 (90)  1 (10) | 3 (100)  3 (100)  0 | 1 (50)  0  0 | 0  0  0 |
| Bedroom  Bedroom adaptation  Medical bed  Sleep system | 2 (20)  8 (80)  7 (70) | 2 (67)  3 (100)  3 (100) | 1 (50)  0  0 | 0  0  0 |
| General home  Home extension  Hoist installed  Specialist seating  Lift installed  Moved to suitable accommodation  Other | 1 (10)  2 (20)  9 (90)  1 (10)  3 (30)  2 (20)^b^ | 2 (67)  3 (100)  3 (100)  2 (67)  0  1 (33)^c^ | 0  1 (50)  0  0  1 (50)  2 (100)^d^ | 0  0  0  0  2 (100)^a^  0 |
| Equipment  Feeding equipment  Oxygen equipment  Sensory toys  Suctioning equipment  Wheelchair  Other | 10 (100)  6 (60)  9 (90)  8 (80)  10(100)  1 (10)^e^ | 3 (100)  1 (33)  3 (100)  3 (100)  3 (100)  0 | 0  0  0  0  0  0 | 0  0  0  0  0  0 |

^a^ Patient went into a nursing home not long after diagnosis as they deteriorated quite quickly.

^b^ In one case, no adaptations were made as parent’s knew that child’s life was limited; in another case, parents were in the process of looking into adaptations.

^c^ Garden adaptation.

^d^ Access adaptation.

^e^ Includes equipment such as: SATs monitor, different seating, profiling bed, and bath aids.

**Supplementary Table 6. Overall symptom burden: Late infantile and early juvenile gene therapy.**

| **Overall symptom burden**  **Frequency, n (%)** | **Late infantile**  **(N=3)** | **Early juvenile**  **(N=3)** |
| --- | --- | --- |
| Mobility  Wheelchair dependent or immobile  Walking with aids  Some issues with walking  No mobility issues | 0  1 (33)  1 (33)  1 (33) | 2 (67)  0  0  1 (33) |
| Learning  Unable to attend school  Attend specialist school  Attend specialist school outreach  Home-schooled  Mainstream school | 0  0  0  0  3 (100) | 0  2 (67)  0  0  1 (33) |
| Speech and communication  Lost all communication  Lost ability to speak  Speech deterioration  No speech issues reported | 0  0  0  3 (100) | 0  0  2 (67)  1 (33) |
| Musculoskeletal  Dystonia  Hip subluxation or dislocation  Hypotonia  Scoliosis  Spasticity or hypertonia  No musculoskeletal issues | 0  0  0  0  0  3 (100) | 0  1 (33)  0  1 (33)  1 (33)  0 |
| Eyesight and hearing  Blindness  Vision difficulties  Deafness  No issues with hearing or eyesight | 0  0  0  3 | 0  1 (33)  0  2 (67) |
| Neurological symptoms  Anxiety or panic  Temperature dysregulation  Peripheral neuropathy  Seizures^a^  Sensory processing issues  Sleep disturbance  Uncontrollable crying  No neurological symptoms | 0  0  1 (33)  0  1 (33)  0  0  2 (67) | 1 (33)^b^  2 (67)^b^  1 (33)^c^  1 (33)^c^  2 (67)^b^  1 (33)^b^  2 (67)^b^  1 (33) |
| Nutrition and eating  Fed by nasogastric tube  Fed by gastronomy tube  Gall bladder issues | 0  0  0 | 0  0  0 |
| Chest and respiratory symptoms  Aspiration  Excess secretions^d^  Frequent chest infections  Frequent colds or runny nose  Ventilation (CPAP or BI PAP)  No chest or respiratory symptoms | 0  0  0  0  0  3 (100) | 0  0  0  0  0  3 (100) |
| Bowels and bladder  Constipation  Urinary retention  Urinary and bowel incontinence  Urinary incontinence only  No issues with bowels or bladder | 0  0  0  0  3 (100) | 1 (33)  0  2 (67)  0  1 (33) |

*CPAP: continuous positive airway pressure; BI PAP: Bilevel Positive Airway Pressure.*

^a^ Suffering from seizures or are on anti-seizure medication.

^b^ Symptom onset before treatment.

^c^ Symptom onset after treatment.

^d^ Suffering from excess secretions or are on anti-secretion medication.
